# miR-125a Suppresses TrxR1 Expression and Is Involved in H_2_O_2_-Induced Oxidative Stress in Endothelial Cells

**DOI:** 10.1155/2018/6140320

**Published:** 2018-08-26

**Authors:** Fangjie Chen, Hong Liu, Jingjing Wu, Yanyan Zhao

**Affiliations:** ^1^Department of Medical Genetics, College of Basic Medical Science, China Medical University, No. 77 Puhe Road, Shenyang North New Area, Shenyang, China; ^2^Department of Clinical Genetics, Shengjing Hospital of China Medical University, No. 36 San Hao Street, Shenyang, China

## Abstract

Thioredoxin reductase (TrxR), an antioxidant enzyme dependent on nicotinamide adenine dinucleotide phosphate, plays a vital role in defense against oxidative stress. However, the role of microRNAs targeting TrxR under oxidative stress has not yet been determined. In this study, we tested the involvement of miRNA-mediated posttranscriptional regulation in H_2_O_2_-induced TrxR1 expression in endothelial cells. Dual luciferase assay combined with expression analysis confirmed that miR-125a suppressed TrxR1 expression by targeting its 3′-UTR. Furthermore, H_2_O_2_ induced TrxR1 expression partly through downregulation of miR-125a. These findings indicate that miRNA-mediated posttranscriptional mechanism is involved in H_2_O_2_-induced TrxR1 expression in endothelial cells, suggesting an important role of miRNAs in the response to oxidative stress.

## 1. Introduction

Growing evidence has shown that oxidative stress may be relevant to a wide range of diseases like cardiovascular disease, tumor, aging, and neurodegenerative disease [1–4]. Indeed, oxidative stress is usually caused by excessive production of reactive oxidative species (ROS) and impaired antioxidant mechanisms [5]. In mammalian cells, the major antioxidant system includes the superoxide dismutase (SOD), glutathione peroxidase (Gpx), catalase (CAT), and thioredoxin system [6, 7]. Well-functioning antioxidant systems are essential for redox homeostasis of cells.

The thioredoxin system comprises thioredoxin (Trx), Trx reductase (TrxR), and NADPH. In this system, TrxR acts in regulating cellular oxidation reduction and protecting cells from oxidative damage by keeping Trx in a reduced state. In addition, TrxRs sustain versatile cellular functions including cell growth, apoptosis, and differentiation [8, 9]. In mammals, there are three identified TrxRs: TrxR1 in the cytoplasm, TrxR2 in mitochondria, and testis-specific isoform TrxR3. TrxR1 is present in most tissues and is denoted as the main and predominant TrxR of the three. Aberrant TrxR1 is found in the development of cardiovascular diseases. TrxR1 mRNA was significantly increased in monocytes of hypertension patients and in atherosclerotic plaques [10, 11], and the serum TrxR activity was significantly increased in coronary artery disease [12]. These data provide empirical evidence that TrxR1 is involved in the development of cardiovascular diseases. To date, the molecular mechanism underlying the transcription regulation of TrxR1 has been well investigated. Activation of nuclear transcription factors, including Sp1, Sp3, Oct-1, and NrF2, has proved to be crucial to transactive *TRXR1* [13]. Studies have also suggested that AU-rich elements (AREs) and SECIS in the *TRXR1* 3′-untranslated region (3′-UTR) regulate its mRNA stability [14]. However, no microRNAs (miRNAs) targeting the *TRXR1* 3′-UTR have been reported until now.

MicroRNAs are a family of small noncoding RNAs that modulate gene expression by partially base pairing with 3′-UTR of their targets [15]. Recent evidence showing altered miRNA expression in the setting of oxidative stress suggests their involvement in oxidative stress and antioxidant defense [16]. Computational searching with TargetScan (http://www.targetscan.org/) and PicTar (https://pictar.mdc-berlin.de/) displayed a putative miR-125a binding sequence within the 3′-UTR of TrxR1 (NM_001093771). It provides the possible involvement of miRNAs in the process of *TRXR1* expression. Studies have demonstrated that H_2_O_2_ is commonly used as an inducer of oxidative stress. Therefore, in this study, we identified miRNA targeting the 3′-UTR of *TRXR1* and elucidated its impact on TrxR1 under H_2_O_2_ treatment in endothelial cells.

## 2. Materials and Methods

### 2.1. Vector Constructs

The 3′-UTR of *TRXR1* was amplified by PCR and cloned into a pGL3-promotor vector (Promega) to produce the pGL3-UTR. The vector containing mutations in the miR-125a binding site of the pGL3-UTR is named as pGL3-UTR-mut. The human miR-125a precursor sequence was amplified and inserted into the pcDNA3.1(+) (Invitrogen) to generate miR-125a-expressing plasmid, pmiR125a. The control vector pcDNA3.1(+) was named pmiR-ctrl. DNA sequencing was performed to verify the orientation and authenticity of all of the inserts. The PCR primers for vector construction are listed in Table 1.

### 2.2. Cell Culture and Reagents

Human embryo kidney HEK293 cells and human umbilical vein endothelial cells (HUVECs) were maintained in Dulbecco's modified Eagle's medium (DMEM) (GIBCO), with 10% fetal bovine serum, 100 *μ*g/ml streptomycin, and 100 IU/ml penicillin, at 37°C in a humidified atmosphere with 5% CO_2_. H_2_O_2_ treatment was carried out at concentration of 0, 0.1, 0.25, and 0.5 mmol/L for 24 h, or at 0.25 mmol/L for 0, 2, 6, 12, and 24 h, respectively. In some of the experiments, the HUVECs were stimulated by a transcription inhibitor, actinomycin D (5 *μ*g/ml), before H_2_O_2_ treatment and harvested after a certain time.

### 2.3. Luciferase Reporter Assay

Cells were seeded in 24-well plates and cotransfected with 500 ng of pmiR125a or 100 nmol/L of miR-125a inhibitor (Ambion) and 200 ng of pGL_3_-UTR or pGL_3_-UTR-mut, the pRL-TK plasmid (20 ng) as the internal control. After 48 h of transfection, the Firefly and Renilla luciferase activities were determined using a luminometer (Berthold).

### 2.4. Detection of miR-125a and *TRXR1* mRNA Expression

Total RNA extracts were prepared from treated or untreated cells using Trizol reagent (Invitrogen). For quantitative analysis of miR-125a, 2 *μ*g of RNA was reverse-transcribed using the miRNA-specific stem-loop primer (Table 1). Real-time quantitative PCR using SYBR Green (Takara) was performed on an ABI Prism 7500 Sequence Detection System, and the expression of miR-125a was detected using the 2^−ΔΔCt^ method with U6 as an internal control. For TrxR1 quantitative analysis, real-time PCR was applied, and GAPDH was amplified as a normalization control. The comparative C_t_ method was used to calculate the relative expression level.

### 2.5. Western Blot

Total protein was extracted from cells, and protein concentrations were determined by Bradford assay (Bio-Rad). Equal amounts of protein were separated in 10% SDS-PAGE and then transferred to a PVDF membrane (Sigma-Aldrich) at 4°C. Membranes were subsequently incubated with the anti-TrxR1 antibody (1 : 2000) (Abnova) or GAPDH antibody (1 : 5000) (Kangcheng) as the primary antibody and followed by HRP (1 : 5000) as the secondary antibody. The final detection reaction was performed with enhanced chemiluminescence detection system (Pierce, Rockford, IL) according to the manufacturer's instructions.

### 2.6. Statistics Analysis

All values were expressed as mean ± SD from three independent experiments, and comparisons between quantitative variables were performed using an independent sample *t*-test. *P* < 0.05 was considered to be statistically significant.

## 3. Results

### 3.1. Identification of the miR-125a Target Site in 3′-UTR of *TRXR1*

**Figure 1:**
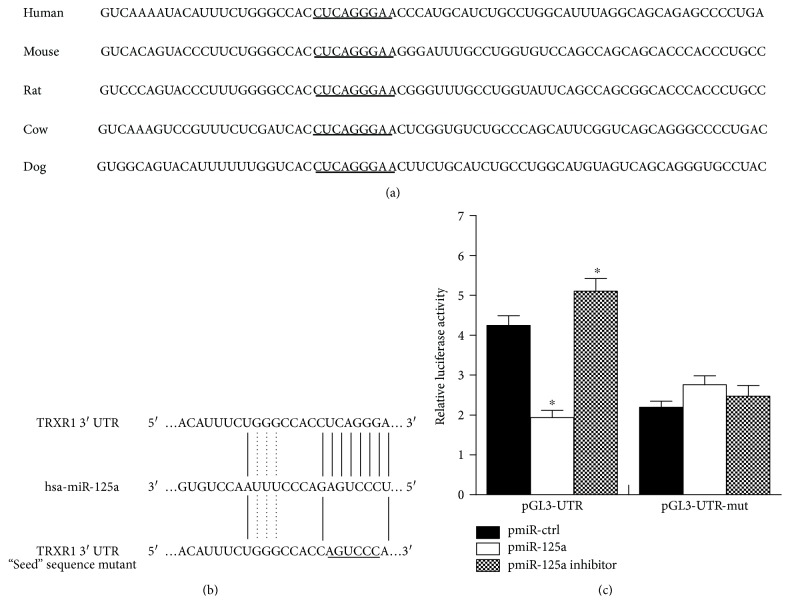
Identification of the miR-125a target site in *TRXR1* 3′-UTR. (a) Conservative sequences of the *TRXR1* 3′-UTR shown in different species. The underlined part is the potential miR-125a binding site. (b) Schematic drawing of miR-125a binding with 3′-UTR of *TRXR1* and its mutant construct. (c) Relative luciferase activities of pGL3-UTR or pGL3-UTR-mut cotransfected with pmiR-ctrl, pmiR-125a, or miR-125a inhibitor in HEK293 cells. ^∗^ *P* < 0.05 compared with pmiR-ctrl.

To find miRNAs that regulated *TRXR1*, we performed bioinformatics analysis using TargetScan and PicTar and found a putative miR-125a target site in *TRXR1* 3′-UTR, which was highly conserved across species (Figures 1(a) and 1(b)). The target site was then confirmed by luciferase assay after cotransfection with pGL3-UTR (or pGL3-UTR-mut) and pmiR-125a (or pmiR-ctrl) in HEK293 cells. As Figure 1(c) shows, pmiR-125a transfection resulted in a marked descent (52%, *p* < 0.05), but the miR-125a inhibitor gave rise to a marked ascent of luciferase activity of pGL3-UTR (23%, *p* < 0.05). However, for the pGL3-UTR-mut, either overexpression or inhibition of miR-125a did not significantly change luciferase activity. According to these data, miR-125a might have bound to the specific sequence in the *TRXR1* 3′UTR.

### 3.2. miR-125a Represses TrxR1 Protein Expression

**Figure 2:**
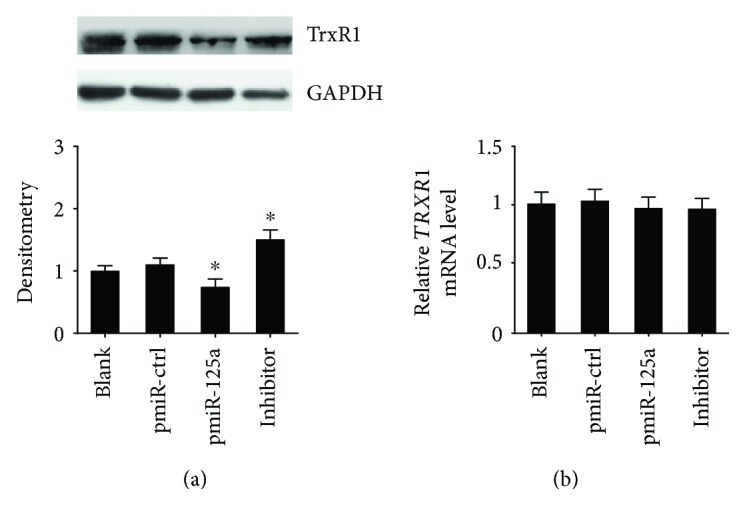
miR-125a suppresses the expression of TrxR1 in HUVECs. (a) Western blot results of TrxR1 for transfected HUVECs (blank, pmiR-ctrl, pmiR-125a, or miR-125a inhibitor treated group, resp.). Data are presented as the ratio of TrxR1 to GAPDH. ^∗^ *P* < 0.05. (b) qRT-PCR results of *TRXR1* mRNA for transfected HUVECs (blank, pmiR-ctrl, pmiR-125a, or miR-125a inhibitor treated group, resp.). ^∗^ *P* < 0.05.

To determine the suppression of TrxR1 expression by miR-125a, we detected both protein and mRNA levels of endogenous TrxR1 in the HUVECs after miR-125a modulation. The Western blot showed that the abundance of the TrxR1 protein significantly reduced (31%, *p* < 0.05) when overexpressing miR-125a and increased by 47% after transfection of miR-125a inhibitor (Figure 2(a)). Moreover, the qRT-PCR result showed a nonsignificant increase of *TRXR1* mRNA compared with the control (Figure 2(b)), suggesting that miR-125a did not induce TrxR1 mRNA degradation. These results suggest that miR-125a may suppress the expression of TrxR1 at the posttranscription level.

### 3.3. H_2_O_2_ Induces TrxR1 Expression but Downregulates miR-125a Expression in Endothelial Cells

**Figure 3:**
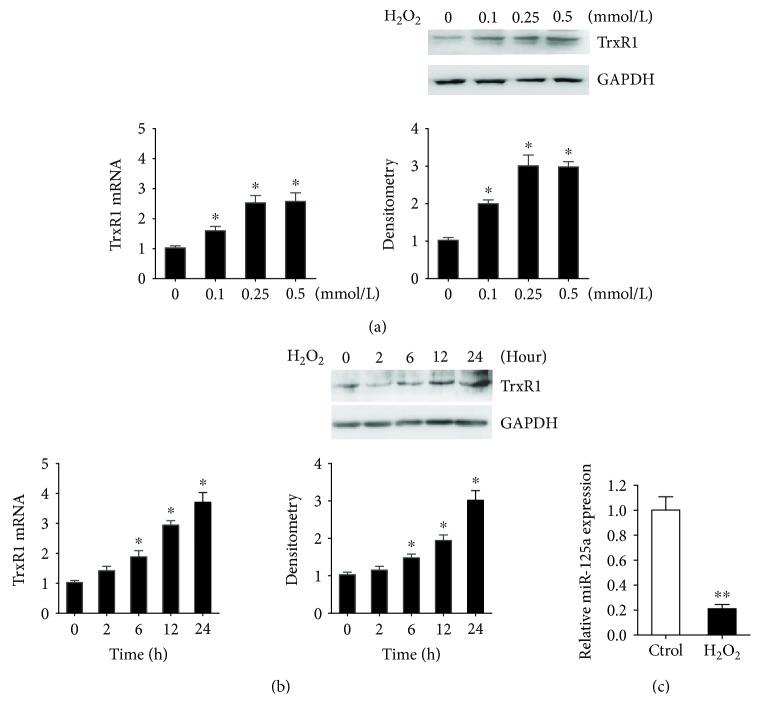
H_2_O_2_ regulates TrxR1 and miR-125a expression in HUVECs. (a) Real-time and immunoblot analysis of TrxR1 in HUVECs treated with H_2_O_2_ (0, 0.1, 0.25, and 0.5 mmol/L). ^∗^ *P* < 0.05. (b) Real-time and immunoblot analysis of TrxR1 in HUVECs treated with 0.25 mmol/L H_2_O_2_ (0, 2, 6, 12, and 24 h). ^∗^ *P* < 0.05. (c) The expression of mature miR-125a in H_2_O_2_-treated HUVECs (0.25 mmol/L) and the control group as determined by real-time PCR. ^∗∗^ *P* < 0.01.

To evaluate the impact of oxidative stress on TrxR1, we examined TrxR1 mRNA and protein in HUVECs exposed to H_2_O_2_ for different dose and time. As shown in Figures 3(a) and 3(b), both mRNA and protein levels were significantly induced by H_2_O_2_ for 2–24 h or at 0.1–0.5 mmol/ml (*p* < 0.05). When cells were exposed to 0.25 mmol/ml H_2_O_2_ for varying amounts of time, a 24 h treatment was required to reach translation peak (3.0-fold over control). Meanwhile, to determine the impact of H_2_O_2_ on miR-125a, real-time PCR was performed. MiR-125a expression was decreased by 80% (*p* < 0.01) in response to the H_2_O_2_ stimulation (0.25 mmol/L) compared with untreated controls (Figure 3(c)). The results obtained from three independent experiments indicate that H_2_O_2_ upregulated TrxR1 mRNA and TrxR1 protein but downregulated miR-125a.

### 3.4. Posttranscriptional Regulation of TrxR1 Expression under Oxidative Stress

**Figure 4:**
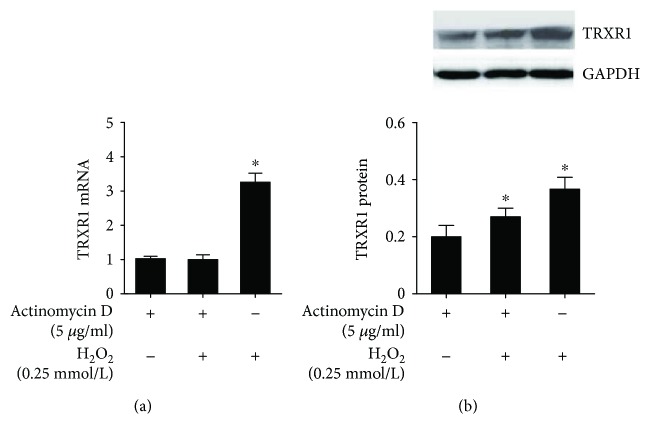
Posttranscriptional regulation occurs in the H_2_O_2_-induced TrxR1 expression. (a) Real-time of TrxR1 mRNA in HUVECs treated with H_2_O_2_ and/or actinomycin D. ^∗^ *P* < 0.05. (b) Immunoblot analysis of TrxR1 protein in HUVECs treated with H_2_O_2_ and/or actinomycin D. ^∗^ *P* < 0.05.

HUVECs were pretreated using actinomycin D (10 ug/ml) for 2 h and then exposed to H_2_O_2_ for an additional 24 h. Complete inhibition of H_2_O_2_-induced TrxR1 mRNA expression was found in the group treated with actinomycin D (Figure 4(a)). However, an evident increase in TrxR1 protein was detected in the H_2_O_2_-stimulated group after treatment with actinomycin D (Figure 4(b)). The level of TrxR1 protein in cells treated with actinomycin D and H_2_O_2_ was ~70% of the TrxR1 level in cells treated with H_2_O_2_. The preceding results suggest the involvement of a posttranscriptional mechanism in H_2_O_2_-induced TrxR1 expression in HUVECs.

### 3.5. Downregulation of miR-125a Is Involved in H_2_O_2_-Induced TrxR1 Expression

**Figure 5:**
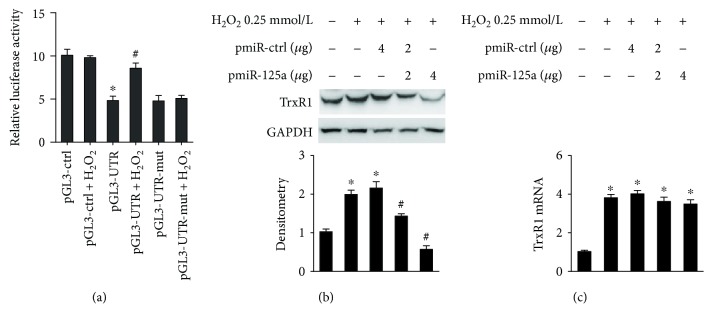
Downregulation of miR-125a is involved in the H_2_O_2_-induced TrxR1 expression. (a) Relative luciferase activity assay after transfection with constructed plasmids and treatment with H_2_O_2_ for 24 h. H_2_O_2_ increased luciferase reporter activity in HUVECs transfected with the PGL3-UTR encoding miR-125a binding site. ^∗^ *P* < 0.05 compared with pGL-ctrl. ^#^ *P* < 0.05 compared with pGL-UTR. (b) Western blot results of TrxR1 in HUVECs treated with pmiR-125a in response to H_2_O_2_ treatment. ^∗^ *P* < 0.05 versus non-H_2_O_2_-stimulated cells. ^#^ *P* < 0.05 versus non-pmiR-125a-treated cells. (c) Real-time analysis of the TrxR1 in HUVECs treated with pmiR-125a in response to H_2_O_2_ treatment. ^∗^ *P* < 0.05 versus non-H_2_O_2_-stimulated cells.

Given the previous results, we speculated that H_2_O_2_ could relieve the miR-125a-mediated TrxR1 suppression by miR-125a inhibition. To confirm this hypothesis, HUVECs were transfected with *TRXR1* 3′-UTR luciferase plasmid containing the binding site of miR-125a. Cells simultaneously exposed to H_2_O_2_ for 24 h reversed the decrease of *TRXR1* 3′UTR-associated luciferase activity compared with non-H_2_O_2_-treated control. There was no marked change of luciferase activity in H_2_O_2_-treated cells transfected with mutant and empty vector control (Figure 5(a)).

To determine whether relief of miR-125a-mediated TrxR1 translational repression was involved in H_2_O_2_-induced TrxR1 protein expression, we transfected cells with an miR-125a expression vector for 48 h and then treated cells to H_2_O_2_ for 24 h. As shown in Figures 5(b) and 5(c), overexpression of miR-125a significantly depressed H_2_O_2_-induced TrxR1 protein expression, but did not decrease TrxR1 transcription compared with H_2_O_2_-treated cells, which were also transfected with the control vector. The preceding results indicate that the relief of miR125a-mediated translational repression of TrxR1 was involved in H_2_O_2_-induced TrxR1 protein expression in HUVECs.

## 4. Discussion

We confirmed that targeting of the *TRXR1* 3′-UTR by miR-125a resulted in *TRXR1* translational suppression. In addition, we found that H_2_O_2_-induced oxidative stress increased the TrxR1 expression but downregulated miR-125a expression. Moreover, H_2_O_2_-induced TrxR1 expression in HUVECs partially involved negation of miR-125a-mediated translational suppression. These results indicate that miR-125a was involved in the H_2_O_2_ *-*induced expression of *TRXR1*, which may be relevant to the regulation of cell responses against oxidative stress in endothelial cells.

To date, extensive research has suggested the functions of miRNAs on oxidative stress-related genes. Eades et al. reported that miR-200a led to Keap1 mRNA degradation by targeting the 3′-UTR of *keap1* [17]. Dong et al. showed that the expression of GSR and POR was suppressed by alcohol-induced miR-214 in liver cells [18]. In addition, miRNAs may be regulated by ROS. Simone et al. revealed that a number of miRNAs including let-7b, miR-15b, and miR-21 increased under ionizing radiation, etoposide, and H_2_O_2_ in human fibroblasts [19]. Thulasingam et al. showed that miR-21 was upregulated while miR-27a decreased under H_2_O_2_-induced stress in PC12 cells [20]. In this work, we verified that miR-125 was directly bound to 3′-UTR of the TrxR1 gene and repressed its endogenous expression, supplying another posttranscriptional regulation mechanism of TrxR1. Moreover, miR-125a expression was significantly downregulated after exposure of endothelial cells to H_2_O_2_. In addition, overexpression of miR-125a significantly depressed H_2_O_2_-induced TrxR1 protein expression. These findings suggest that miR-125a mediating the downregulation of TrxR1 plays an important role in H_2_O_2_-induced oxidative stress in endothelial cells. miR-125a was first identified in the brain tissue of mice by Northern blot in 2002 [21]. Recently, it has been confirmed that miR-125a exerts growth regulation, lipid uptake, and vasomotor homeostasis through targeting p53, oxysterol binding protein-related Protein 9, and endothelin-1 (ET-1) genes [22–24]. Our study confirmed that *TRXR1* was a newly identified target of miR-125a. To our knowledge, miRNAs are always fine-tuning posttranscriptional regulators of target mRNAs in most biological processes, including in cellular responses to redox imbalance [25]. Therefore, we postulated that miR-125a could be a key posttranscriptional regulator in oxidative stress-mediated diseases. When assessing the effect of miR-125a in H_2_O_2_-induced oxidative stress, we found that TrxR1 was markedly increased after H_2_O_2_ treatment, consistent with the results of Furman et al. [11]. However, mir-125a decreased significantly in H_2_O_2_-treated HUVECs. ROS modulating oxidation-sensitive signaling pathways and transcription factors is the common mechanism responsible for ROS-mediated genes. Further research should be performed to elucidate the underlying mechanism.

An imbalance between oxidative stress and the antioxidative system in endothelial cells is generally considered to be the common mechanism causing cardiovascular diseases. To keep redox-balanced conditions, cells always protect themselves from oxidative injury through activation of the antioxidant system [26, 27]. H_2_O_2_-induced TrxR1 expression results in further scavenging of ROS. Furthermore, downregulated miR-125a in the setting of oxidative stress relieves miR-125a-mediated translational repression of TrxR1, which thereby functions better in antioxidant defense.

In conclusion, miR-125a targeted *TRXR1* 3′UTR and resulted in downregulation of endogenous *TRXR1* expression in HUVECs. Moreover, miR-125a was involved in H_2_O_2_-induced oxidative stress. These results indicate that miR-125a may play a vital role in antioxidant defense via posttranscriptional regulating *TRXR1* and may be a new target to regulate endothelial function.

## Figures and Tables

**Table 1:** Primers for vector construction and quantitative PCR.

Name	Sequence
pGL3-3′UTR-forward	5′-CATTTGCAATGGAAAACACG-3′
pGL3-3′UTR-reverse	5′-TGCCTCAATTGCTCTCTCCT-3′
Mutant-forward	5′-TACATTTCTGGGCCACCTCAGTCA ACCCATGCA T-3′
Mutant-reverse	5′-CAGGCAGATGCATGGGTTGACTGAGGTGGCCCAG-3′
pmiR-125a-forward	5′-TCCCTCTTATTCTGGCTTTC-3′
pmiR-125a-reverse	5′-CATCCCAACAAACATCTGG-3′
miR-125a-forward	5′-ACACTCCAGCTATATCCCTGAGACCCTTTA-3′
miR-125a-reverse	5′-GGTGTCGTGGAGTCGGC-3′
U6-forward	5′-CTCGCTTCGGCAGCACA-3′
U6-reverse	5′-AACGCTTCACGAATTTGCGT-3′
miR-125a RT primer	5′-CTCAACTGGTGTCGTGGAGTCGGCAATTCAGTTGAGTCACTGGT-3′

## Data Availability

The data used to support the findings of this study are included within the article.

## Abbreviations

TrxR: Thioredoxin reductase
miRNA: MicroRNA
UTR: Untranslated region
H_2_O_2_: Hydrogen peroxide.

## Supplementary Materials

Supplementary Materials Figure S: The effect of pmiR-125a on mature miR-125a expression and the verified target protein P53.
