# Supplementary material for: miR-125a Suppresses TrxR1 Expression and Is Involved in H2O2-Induced Oxidative Stress in Endothelial Cells
Source: J Immunol Res. 2018 Aug 26;2018:6140320. doi: 10.1155/2018/6140320 (PMC6129346; doi:10.1155/2018/6140320)
Supplement: Supplementary Materials — Figure S: The effect of pmiR-125a on mature miR-125a expression and the verified target protein P53. [file 6140320.f1.pdf]

Supplementary material:

To validate the effects of pmiR-125a, HEK293 cells were transfected with pmiR-ctrl and pmiR-125a for 48 h. Real-time PCR was performed to detect the level of miR-125a. Western blot was carried out to examine p53 protein, which Zhang *et al.* verified as the target for miR-125a [1]. As expected, transfection of pmiR-125a resulted in a substantial increase in miR-125a in comparison with pmiR-ctrl-transfected cells (Figure S). Over-expressing miR-125a after transfection of pmiR-125a markedly reduced the endogenous P53 protein levels of HEK293 cells, and miR-125a inhibition resulted in an increase of P53. The results show that pmiR-125a could efficiently express microRNA-125a.

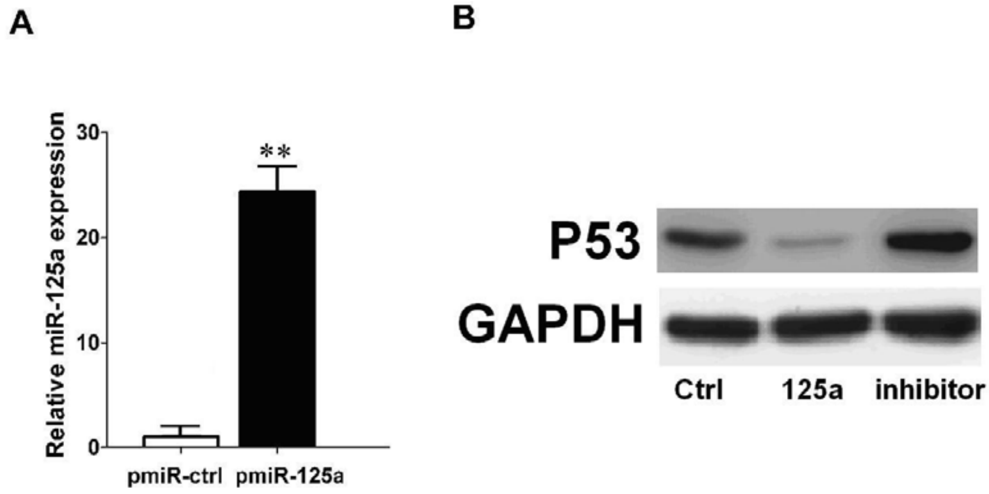

Fig. S. The effect of pmiR-125a on mature miR-125a expression and the verified target protein P53. (A) Overexpression of miR-125a by transfection of pmiR-125a, but not the pmiR-ctrl, resulted in increased miR-125a levels as measured by real-time PCR. (B) P53 as a positive control could be suppressed by pmiR-125a, but up-regulated after transfection with miR-125a inhibitor.
